# Recent Progress in Transdermal Nanocarriers and Their Surface Modifications

**DOI:** 10.3390/molecules26113093

**Published:** 2021-05-21

**Authors:** Zhixi Yu, Xinxian Meng, Shunuo Zhang, Yunsheng Chen, Zheng Zhang, Yixin Zhang

**Affiliations:** 1 Department of Plastic and Reconstructive Surgery, Shanghai Ninth People’s Hospital, School of Medicine, Shanghai Jiao Tong University, 639 Zhizaoju Rd, Shanghai 200011, China; caroleyu@126.com (Z.Y.); m_sharon@sina.com (X.M.); feltfans2013@163.com (S.Z.); 2 Shanghai National Engineering Research Center for Nanotechnology, 245 Jiachuan Road, Shanghai 200237, China

**Keywords:** transdermal drug delivery, transdermal nanocarrier, surface modification, enhanced penetration efficiency, controlled release, targeting delivery

## Abstract

Transdermal drug delivery system (TDDS) is an attractive method for drug delivery with convenient application, less first-pass effect, and fewer systemic side effects. Among all generations of TDDS, transdermal nanocarriers show the greatest clinical potential because of their non-invasive properties and high drug delivery efficiency. However, it is still difficult to design optimal transdermal nanocarriers to overcome the skin barrier, control drug release, and achieve targeting. Hence, surface modification becomes a promising strategy to optimize and functionalize the transdermal nanocarriers with enhanced penetration efficiency, controlled drug release profile, and targeting drug delivery. Therefore, this review summarizes the developed transdermal nanocarriers with their transdermal mechanism, and focuses on the surface modification strategies via their different functions.

## 1. Introduction

Transdermal drug delivery systems (TDDS) have been developed as an attractive drug administration method in clinical dermatology [1]. Compared to the conventional oral route, intravenous, or subcutaneous injection, the TDDS provide an easier application without the first-pass effect and systemic side effects [2]. Nowadays, TDDS are widely applied in treatment for many dermatological diseases, such as psoriasis, contact dermatitis, and skin cancer. Moreover, TDDS is also suitable for long-term administration, especially for insulin delivery and some analgesic agents [3]. However, TDDS need to conquer the skin barriers, including stratum corneum (SC), surrounding lipid bilayer, and dermal tissue [4].

Therefore, great efforts have been made to achieve a better transdermal delivery efficiency during the past decades. Many transdermal delivery enhancement technologies have been developed from the first-generation to the fourth-generation, such as iontophoresis, electroporation, ultrasound, microneedles, and most recently, the nanocarriers [5]. Among them, nanocarriers show the greatest potentials in clinical applications, because of their independence on extra equipment, low skin irritation, and no damage on the intact skin barrier. In addition, nanocarriers can encapsulate macromolecules and hydrophilic agents such as non-steroidal drugs, photosensitizers, and some chemotherapy agents, as well as increase the drug stability and retention [6,7,8,9].

Various nanocarriers have been developed and investigated as useful TDDS. By optimizing the components and formulation, the nanocarriers can achieve ideal morphological and physico-chemical characteristics (such as size and surface charge). Although they display the potentials in transdermal delivery, their therapeutic applications suffer from their limited penetration ability, drug encapsulation and release, and so on [10]. Recently, based on the well-developed nanocarriers, many studies concentrated on surface modification to further improve their therapeutic applications. The flexible structures and exposed functional groups provide the opportunity for this procedure.

Herein, this review aims to present recent progress on different transdermal nanocarriers as well as their functionalization with different surface modifications. Furthermore, the functional transdermal nanocarriers can overcome the barriers in transdermal delivery, and their surface modification strategies provide feasibility ideas to promote their clinical applications.

## 2. Overview of Transdermal Drug Nanocarriers

**Figure 1:**
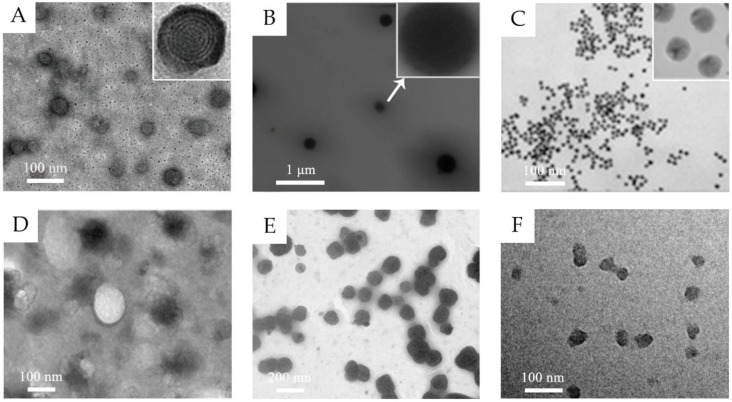
(**A**) TEM images of 5-aminolevulinic acid-loaded ethosomal vesicles. Adapted from Reference [8] with permission of The Royal Society of Chemistry. (**B**) TEM images of triptolide-loaded nanostructured lipid carriers (NLC). Adapted from Reference [11] with permission. (**C**) TEM images of the citrate-capped gold nanoparticles. Adapted from Reference [12] with permission. Copyright (2017) American Chemical Society. (**D**) TEM images of silk fibroin nanoparticles. Reproduced from Reference [13] Copyright (2018), with permission from Elsevier. (**E**) TEM images of water-in-oil (w/o) nanoemulsions of caffeine. Reproduced from Reference [14]. Copyright (2010), with permission from Elsevier. (**F**) TEM images of hyaluronic acid nanogels. Reproduced from Reference [15]. Copyright (2015), with permission from Elsevier.

In the past decades, lots of nanocarriers have been developed and applied for transdermal drug delivery. Their structure and transdermal delivery mechanism vary with different components. The most investigated transdermal nanocarriers, including lipid-based nanovesicles, lipid nanoparticles, polymeric nanoparticles, some inorganic nanoparticles, and others, are summarized below (Table 1 and some transmission electron microscopy (TEM) images shown in Figure 1).

### 2.1. Lipid-Based Nanovesicles

Lipid-based nanovesicles are defined as spherical vesicles with one or more lipid bilayers and aqueous inner core, in which hydrophilic drugs can be encapsulated in internal core and hydrophobic drugs can be inserted in external lipid bilayers. Liposomes, the classic and most mature lipid-based nanovesicles, are generally formed with phospholipid molecules and cholesterol as a stabilizer [35]. The phospholipid component could interact with the lipids of the SC, namely fusion mechanism, and thus achieve the transdermal effect. Some deformable liposomes are also reported to penetrate through SC with their intact structure [16]. However, its penetration in the deeper skin layer is limited and the accumulation was mainly observed in the epidermis because of its relatively low fluidity [36]. Therefore, great efforts have been made to improve the skin penetration.

Ethosomes and transfersomes are the most investigated innovative lipid-based nanovesicles, with increased softness, deformability, and elasticity [37]. Ethosomes, composed of phospholipid and ethanol, are generally formed as multilamellar nanovesicles. Ethanol can increase the fluidity of phospholipid bilayers, disrupts the membrane barrier of SC, and thus enhances the penetration ability [17]. Transfersomes are composed of phospholipid and edge activators (such as Tween 80, Span 80, and sodium cholate). The edge activators, as the membrane-softening agent, endow transfersomes with remarkable flexibility and deformability for passing through the narrow pores [18].

Some other lipid-based nanovesicles are also developed with different compositions. Niosomes are synthesized with cholesterol and nonionic surfactants, which replace the phospholipid. The nonionic surfactants enhance the drug encapsulation efficiency and cholesterol increases the rigidity and stability [19]. Glycerosomes, as the term implies, are composed of glycerol, which serves as the same role of edge activators in transfersomes. The glycerol improves the elasticity and deformability of the vesicle with a dose-dependent effect [20]. Terpenes are introduced to form the invasomes because they can disrupt the SC lipid structure and increase the membrane elasticity at a low concentration as 1% *w/v* [21].

### 2.2. Lipid Nanoparticles

Different from lipid-based nanovesicles, lipid nanoparticles are solid particles, mainly encapsulating drug molecules in a non-aqueous core. They include solid lipid nanoparticles (SLN), composed of solid lipids, and nanostructured lipid carriers (NLC), as the second generation, composed of solid and liquid lipids. Compared to SLNs, liquid lipids in NLCs avoid recrystallization of solid lipids and thus increase the stability. Some surfactants are introduced to reduce the interfacial tension between the aqueous phase and hydrophobic lipid structure and to improve the formulation stability [38].

The transdermal delivery mechanism of lipid nanoparticles remains unclear. The possible mechanisms are as follows: the lipid nanoparticles demonstrate skin adhesive property and form a mono-layer lipid film, which results in the “occlusion effect”, to avoid the water evaporation, enlarge the inter-keratinocyte gap, and thus enhance the drug penetration [22]. It is also reported that the lipid components and incorporated surfactant could disrupt skin structure and increase the intercellular space by their interaction with skin lipid layer, especially in SC. Gu et al. observed the skin structure after being treated with triptolide-loaded lipid nanoparticles [11]. The SEM images and histopathological analysis demonstrated the inflated SC, loose texture, and dilated epidermis, indicating the interaction between lipid nanoparticles and skin.

### 2.3. Polymeric Nanoparticles

Polymeric nanoparticles can be prepared by both natural polymers and synthetic polymers. The natural polymers, such as chitosan, gelatin, and albumin, are easily biodegradable and biocompatible. Chitosan, one of the most frequently used natural materials, is positively charged, which is suitable for encapsulating negative-charged drugs via electrostatic interaction and facilitates the cellular internalization [39]. Meanwhile, the functional groups of chitosan provide great potential for further surface modification [40]. Compared to natural polymers, the synthetic polymers show better purity and consistency. Synthetic polymers are biodegradable, such as polylactides and poly (lactic-*co*-glycolic acid) (PLGA) copolymers, or non-biodegradable, like poly (methyl methacrylate) and polyacrylates. The nanoparticles formed from synthetic polymers are frequently used for delivering the hydrophobic drugs [39].

The size of polymeric nanoparticles is generally too large to pass through the SC, where they can form as a drug reservoir and create a drug concentration gradient to enhance the drug permeation [22]. Tomoda et al. introduced the iontophoresis to enhance the skin penetration of negatively charged PLGA nanoparticles [41]. Takeuchi et al. developed positively charged chitosan-coated PLGA nanoparticles for iontophoretic transdermal delivery of positively charged drugs and observed that the nanoparticles penetrated through the follicle pathway [23]. Recently, Takeuchi et al. synthesized 40-nm silk fibroin nanoparticles which can reach the deeper dermis through both paracellular route and hair-follicle route [13]. It is indicated that polymeric nanoparticles smaller than a certain size can penetrate through the epidermal barrier.

### 2.4. Inorganic Nanoparticles

Inorganic nanostructures are widely applied in drug delivery for cancer therapy with their probable bio-imaging and phototherapy potential. Some nanoparticles with positive charge, high surface lipophilicity, and small size have transdermal capability by passively penetrating SC [2]. Because of their great stability and high potential for surface functionalization, some studies have also evaluated the transdermal drug delivery efficiency of some inorganic nanoparticles.

Gold nanoparticles are well explored as TDDS, because of their low cytotoxicity and controllable particle size [42]. As reported, the skin penetration amount and rate increase with the decreasing particle size; moreover, the accumulation of 15-nm size particles are observed in deep dermis [24]. Furthermore, the surface ligands bind with the gold nanoparticles with Au-S bonding, which means various ligands could be conveniently conjugated on the surface of particles by pre-thiolation [12]. This specific characteristic of gold nanoparticles enables the application in gene delivery. Zheng et al. conjugated gold nanoparticles with thiolated epidermal growth factor receptor (EGFR) siRNA duplexes (SNA-NCs) via Au-S bonding [25]. The SNA-NCs penetrated through whole skin layer and successfully abolished EGFR expression in mouse model.

Some other metallic particles are also observed for transdermal application. Fe_3_O_4_ nanoparticles with a pH-sensitive amide bond successfully penetrate into deeper dermis via transfollicular route [26]. Photothermal CuS nanoparticles with near-infrared irritation can induce localized thermal ablation of SC and facilitate the penetration of particles [27].

### 2.5. Other Nanocarriers

Dendrimers are high-branched polymeric nanocarriers used as TDDS for transport hydrophobic agents and macromolecules, such as photosensitizers and chemotherapeutic agents. Conventional dendrimers with composition of poly (amidoamine) could not effectively cross the skin barrier but show penetration enhancer potential because of the interaction with skin lipid bilayers [28]. Recently, some studies concentrate on the size and surface charge of dendrimers and then developed the second-generation dendrimers with smaller size and skin penetration capability [43].

Micelles, also one type of polymeric nanocarriers, are composed of amphiphilic polymers or surfactants agglomerating in an aqueous medium [29]. Their flexible formulation endows micelles with drug co-delivery ability. Generally, the lipophilic drugs are loaded in core, while hydrophilic drugs are located mainly in the shell [30]. As TDDS, the micelles can increase the skin penetration by improving the water solubility of drugs. The polymer components are blocked by intact SC, and thus have less improvement for low-permeability drugs [31].

Nanoemulsions (NE) are dispersions of water and oil stabilized by surfactants, forming nanometric droplets of oil in water (O/W), water in oil (W/O), and bi-continuous emulsions. These different systems are suitable for delivering both hydrophobic and hydrophilic drugs. The oil phase and surfactants can disrupt the skin lipid bilayers and SC and enhance the drug permeation. Furthermore, the unique bi-phase structure provides excellent solubility for both liposoluble and water-soluble drugs, which leads to an increased drug concentration gradient for better penetration [22]. Shakeel et al. prepared W/O nanoemulsions of hydrophilic caffeine and 5-fluorouracil for transdermal anti-cancer therapy [14,44]. The W/O nanoemulsions significantly increased the drug permeation through para-cellular transportation. The limitation of nanoemulsions is long-term stability with the risk of Ostwald ripening, and overdosing surfactants may result in additional cytotoxicity [45]. Therefore, an ideal formulation with a suitable surfactant ratio still needs to be developed.

Nanogels are chemically or physically cross-linked polymers in nanosize swelling in medium. The cross-linked network structure demonstrates unique stimuli-responsive nature for drug loading and modulated releasing [15]. Nanogels could enhance the drug penetration mainly by following two aspects. Firstly, the stable nanocarrier dispersions in medium prolong the topical contact duration and form a localized high concentration of loaded drugs. Secondly, cationic polymers could be introduced in the network structure to interact with negatively charged keratinocytes [32]. Jayakumar et al. developed curcumin and 5-fluorouracil-loaded chitin nanogels for melanoma treatment [33,34]. The drug retention in the dermis of cationically charged chitin nanogels increased 4- to 5-fold as compared to the control solution. As observed in histological studies, the thinner keratin layer and fragmentation of SC revealed the interaction of chitin and epidermis.

## 3. Surface Modifications and Their Functions

Although various types of nanocarriers have been developed as TDDS, they still face some challenges, such as limited drug penetration efficiency, burst release, and lack of targeting. Recently, some studies focused on surface modifications to improve the transdermal nanocarriers to overcome their limitations (Table 2).

### 3.1. Enhanced Penetration Efficiency

The penetration efficiency, including drug penetration depth and drug permeation amount, is the most critical parameter of the transdermal nanocarriers [65]. The drug transdermal diffusion pathways are mainly divided into two pathways: transappendageal pathway and transepidermal pathway [4]. The transappendageal pathway is known as a shunt route, because the presence of skin appendages, such as hair follicles and sweat glands, provide natural openings for drug penetration [66]. The transepidermal pathway, including intercellular pathway, is a tortous route through intercellular bilayered matrix, and the transcellular pathway is where drugs diffuse directly through corneocytes. Although the transappendageal pathway has natural opening with high penetration efficiency, it constitutes only 0.1% of skin surface area [4,22], and even does not exist in some skin lesions and scars. Therefore, the improvement generally concentrates on intercellular pathway and transcellular pathway. The current main strategies are divided into two aspects, disturbing cell membrane and cell junction with lipid components and some surface surfactants, or increasing cell uptake with cationic ligands and cell penetrating agents.

Oleic acid is generally used as penetration enhancer for drug passive transdermal penetration by increasing the fluidity of skin lipids [67]. Pando et al. investigated the effect of oleic and linoleic acids on the niosomes entrapping resveratrol, a low water-soluble and photosensitive drug [46]. The results indicated the oleic and linoleic acids can enhance the penetration of poorly water-soluble drugs. Punit et al. developed polymeric bilayered nanoparticles with PLGA inner core and chitosan as an outer coat for simultaneous delivery of spantide II (SP) and ketoprofen (KP) for inflammatory skin disorders [47]. The nanoparticles are modified with succinimidyl glutarate ester of PEGylated oleic acid by the formed covalent amide bond between chitosan and PEG derivative. The in vitro penetration depth of the modified nanoparticles has been enhanced up to 240 μm and the cumulative amount after 24 h of SP and KP were 4.1- and 3.1-fold higher, respectively.

Ceramides, the major component of SC, has been investigated for improving the skin penetration. Abdelgawad et al. doped the ceramide into a vesicular phospholipid system, called “cerosomes”, which entrapped the tazarotene for the psoriasis treatment [48]. The in vitro experiments revealed that the “cerosomes” increased the drug deposition in the skin compared to the marketed product Acnitaz^®^. They also evaluated the clinical efficiency of the tazarotene loaded “cerosomes” and Acnitaz^®^. After 8 weeks of treatment, the lesions tropically treated with “cerosomes” were significantly reduced and had marked improvement. It should be noted that, as reported, the presence of ceramide resulted in a shape transformation from spherical to the tubular morphology, which still needs more research.

Some polymers have also been used to enhance the penetration and permeation of transdermal nanocarriers. Poly (ethylene glycol) (PEG), one of the most commonly used polymers, shows great improvement in the transdermal penetration efficiency. As Mahmoud et al. reported, the PEG coating could prevent the aggregation by providing steric repulsion [68]. Sunoqrot et al. have further investigated PEG with different termini for the melanin-mimetic polydopamine nanoparticles (PDA NPs) [49]. The PDA NP itself accumulated in the Strat-M membrane, while the PEGylated NPs could penetrate to the dermis. Among different various termini on the NPs surface, the anionic PEGylated NPs obtained up to 78% drug accumulation, with great colloidal stability. Hiranphinyophat et al. introduced a polymer, poly (2-isopropoxy-2-oxo-1,3,2-dioxaphospholane) (PIPP), as a surface modifier for the cellulose nanocrystal (CNC)-stabilized emulsions [50]. The PIPP bound with CNC via ring opening polymerization and the PIPP increased the surface hydrophobicity, emulsifying efficiency, and the particle stabilization. The in vitro study demonstrated that the PIPP-modified particle-stabilized emulsions could penetrate through all skin layers with increasing drug accumulation.

Cell-penetrating peptide (CPP), a kind of short, water soluble, cationic peptide, with cell penetration ability, was recently investigated as the surface modifier. Several studies demonstrated its improvement in skin penetration and permeation because it could destabilize SC, interact with cell membrane via electrostatic interaction, and increase cell uptake [69]. Experimental results reveal that the content of cationic amino acids is crucial for CPP internalization and the arginine residues have the greatest impact on internalization. The 7–15 residues of polyarginine provide most optimal improvement [70]. Kwon et al. conjugated the CPPs to DOPC liposomes via a thiol-maleimide reaction for delivering *Polygonum aviculare* extract [51]. Compared to the typical liposomes, this system improved skin penetration ability and increased the cell uptake. Wang et al. reported a CPP conjugated lipid vesicle with SPDP cross-linker, demonstrating a higher storage stability, increased drug delivery amount and enhanced penetration ability [52]. Jiang et al. have synthesized CPP-modified transfersomes for delivering the paclitaxel, a chemotherapy drug of melanoma [53]. With the CPP modification, they successfully sent the paclitaxel to the xenografted tumor tissues via transdermal route in a mouse model.

### 3.2. Controlled Release

Traditional topical medicines, such as topical corticosteroids, generally result in local side effects with long-term application and could lead to the systemic side effects with an extreme overdose and over-absorption [71]. With development of nanocarrier encapsulation structure, some studies concentrated on the transdermal controlled release to overcome the mentioned problems. The reported controlled release system of transdermal nanocarriers could be divided into two release patterns, the sustained release and the activated modulated release.

In sustained drug release, the drug release is prolonged over a period of time, which provides a decreased application frequency, a prolonged treatment effect, and a relatively stable drug concentration. Silva et al. conjugated the oleic acid on the polymeric nanoparticles to deliver betamethasone [54]. In vitro drug release study demonstrated a burst release of 50% loaded corticoid in the initial 10 h with a following controlled release lasting 72 h. In addition, with encapsulation in the nanoparticles, the drug degradation was reduced and the skin penetration was enhanced by oleic acid as reported before.

Pashirova et al. synthesized a type of surfactant containing natural moiety quinuclidine and 1,4-diazabicyclo [2.2.2]octane (Dabco) presenting antimicrobial activity [55]. They modified liposomal system with this surfactant and reported a more sustained release of loaded drug with 50% of release in 12 h. The embedded surfactants increased the rigidity and stability of the liposomal structure, which provided the controlled release ability, with significant influence on the skin penetration ability.

Chitosan, a biodegradable and biocompatible polymer with mucoadhesive property, is also an ideal surface modifier for increasing the surface rigidity for nanocarriers [40]. Shekh et al. conjugated the oxidized chitosan (OC) on electrospun polyacrylonitrile nanofibers (PAN NFs) with ethylene diamine (EDA) as cross-linker for the transdermal delivery of acyclovir (ACY) [56]. The surface anchored OC provided abundant unreacted CHO for facilitating the binding of drug molecules via Schiff’s reaction. In vitro drug release in PBS presented that the bared PAN NFs released over 90% of ACY in 5–7 h, while the PAN-EDA-OC NFs released 50% of ACY with a controlled rate in 9 h.

In activated modulated release, the drug release is triggered by certain physical or chemical process, meaning that, different from the sustained drug release pattern, activated modulated release requires a response element. The temperature difference is one of the most notable features of skin barrier, therefore, Fujimoto et al. developed a temperature-responsive liposome (TR liposome) by incorporating a thermosensitive polymer, named poly-*N*-isopropylacrylamide (PNIPAAm) [57]. The drug release and skin permeation began at 37 °C and presented a temperature-dependent effect. Although the PNIPAAm incorporation led to a larger particle size and slightly decreased the skin absorption, with the assistance of fractional laser irradiation, both drug release and transdermal permeation were enhanced.

Rancan et al. introduced a linear thermosensitive polyglycerol (tPG) to dendritic polyglycerol (dPG)-based nanogels for inflammatory skin diseases [58]. The skin penetration in disrupted skin samples, which mimicked the inflammatory situation, was enhanced but still mainly limited in the SC. After irritation with infrared lamp to achieve 40 °C of topical temperature, the drug release was triggered and the tissue penetration could reach the deeper dermis.

Photothermal nanocarriers also show the potential for triggered drug release. Zhang et al. synthesized a molybdenum disulfide (MoS_2_) nanoparticle modified with cationic hydroxyethyl cellulose (JR400) [59]. The JR400 provided the stability of drug encapsulation without irritation and reduced the cytotoxicity of MoS_2_ NPs. JR400-MoS_2_ NPs irritated by near-infrared 808 nm laser demonstrated a significant photothermal effect and an increased drug release. However, the synthesized nanoparticles themselves do not have transdermal properties, and thus the application of this drug delivery system is limited.

### 3.3. Targeting Drug Delivery

As long-term application of many chemotherapeutic agents and steroid hormones has severe local and even systemic side effects, development of tissue or cell targeting drug delivery is critical. The nanocarrier systems show great potential in targeting delivery because of their flexible surface modifications [72]. Different from the intravenous administration, the transdermal route bypasses the vascular system, which leads to absence of enhanced permeability and retention (EPR, a passive targeting delivery) effect. Therefore, the improvement of the transdermal nanocarriers concentrates on the active targeting strategies, such as some ligands for targeting specific receptors [73].

Hyaluronic acid (HA), a biocompatible polymer with transdermal effect, also could specifically bind to CD44 receptors, which overexpress in various cancer cells [74]. Kong et al. modified the niosomes with HA derivatives to develop the transdermal tumor targeting nanocarrier [60]. They reported an enhanced endocytosed amount of nanocarrier by 4T1 cells with the presence of HA derivatives. Beack et al. conjugated HA to the chlorin e6 (Ce6)-carbon dot (Cdot) by EDC/NHS cross-linking for the melanoma photodynamic therapy [61]. The Cdot-Ce6-HA conjugate demonstrated improved tissue penetration and specific cancerous skin accumulation. With laser irritation, the tumor growth of subcutaneous grafted B16F10 cells was significantly suppressed, while the tumor volume of free Ce6 group and Cdot-Ce6 group increased.

Epidermal growth factor (EGF), a specific ligand for epidermal growth factor receptor (EGFR), which is also overexpressed in cancer cells, showed its potential for target delivery. Ruan et al. designed a skin-penetrating and cell-entering peptide (SPACE) nanocarrier modified with EGF for siRNA delivery to melanoma cells [62]. The in vitro experiments indicated that the EGF gave the melanoma cell targeting ability without influence on the tissue penetration function of SPACE.

In recent years, some other ligands are screened for selective cell targeting strategies. Huang et al. chose the BQ-788, an antagonist selectively binding to the endothelin ET_B_ receptors for melanocytes, to functionalize the ZnO quantum dots (ZnO QDs), for transdermal delivery of a TYR inhibitor [63]. They reported a specific cell uptake by health melanocytes and no observed bind on the human keratinocyte line. Meanwhile, the BQ-788/ZnO QDs in vitro delivered the TYR inhibitor. Gu et al. synthesized a high-affinity ligand for integrin αvβ3 (named PEP), an overexpressed cell adhesion protein in cancer cells [64]. They constructed an *Escherichia coli* (*E. coli*)-derived outer membrane vesicle (TEVs) modified with PEP for transdermal and tumor-targeting delivery. Both in vitro and in vivo experiments showed a specific accumulation of PEP-TEVs in the melanoma cells. As reported, the PEP-TEVs delivered the plasmid-DNA and ICG efficiently, indicating its potential as a multi-functional vector.

## 4. Conclusions and Outlook

In this review, various transdermal nanocarriers are introduced with their physico-chemical characteristics, applications as well as delivery mechanism. Based on the present research, the lipophilic components can interact with skin lipid layer, disrupt skin structure, increase the intercellular space, and thus facilitate the skin penetration. Both nanovesicles and solid nanoparticles can serve as TDDS. The bilayer membrane structure endows nanovesicles with remarkable deformability and elasticity, while non-aqueous core provides solid nanoparticles with better stability. In general, nanovesicles encapsulate hydrophilic drugs in aqueous interior cores and load hydrophobic drugs in external lipid bilayers. On the contrary, solid nanoparticles shield hydrophobic drugs in lipid core and absorb hydrophilic drugs in outer aqueous phase. Moreover, smaller size is recommended for higher specific surface area, which probably increases non-specific cell uptake and facilitates transcellular penetration. However, the correlation of size and penetration efficiency is not absolute, and that encapsulation efficiency of drugs depends on the size of nanoparticles. In addition, the influence of surface charge still remains controversial. Although the positive surface charge is generally considered to promote the skin penetration via enhanced electrostatic interaction with the cell membrane, some researches have investigated a higher transdermal efficiency the negatively charged nanoparticles. It indicates that excessive cell internalization and skin retention will result in reduced skin penetration. Therefore, the ideal range of particle size and surface charge needs to be explored.

Furthermore, their surface modification strategies are also reviewed to overcome the challenges of limited penetration, uncontrolled release, and lack of targeting. As mentioned above, we summarized the investigated surface modifiers according to their functions. Conjugation of penetration enhancer increases the transdermal penetrability and thus allows therapeutic agents to penetrate thick skin tissue in dermatitis and dense fibrous tissue in scars. The realization of both sustained and activated modulated drug release makes it possible to design the transdermal nanocarrier according to optimal tissue drug concentration. Moreover, within selected specific ligands for target receptors, the transdermal nanocarriers achieve the active drug targeting delivery capability, which is crucial especially for skin cancer treatment. These surface modifications improve and enlarge the clinical application of transdermal nanocarriers.

Recently, systemic delivery via transdermal route shows great potential, especially for insulin, vaccine, and analgesic agents, which can be only achieved by microneedle systems at present. However, the existing transdermal nanocarriers are still limited in systemic administration. To achieve a systemic delivery, deeper tissue penetration, transvascular transport ability, and stable release rate are necessary. In addition, there are inadequate in vivo studies about clear transdermal delivery mechanism and safety of long-term administration, which are critical for further clinical translation. Moreover, ideal formulation, high synthetic reproducibility, and good stability for storage are also necessary for pharmaceutical aspect.

## Figures and Tables

**Table 1:** Summary of typical components, structure, and transdermal mechanisms of transdermal nanocarriers.

Classification		Typical Components	Structure	Transdermal Delivery Mechanism	Ref.
Lipid-based nanovesicles	Liposomes	Phospholipid, cholesterol	Spherical vesicles with one or more lipid bilayers and aqueous inner core	The phospholipid component interacts with the lipids of the SC.	[16]
	Ethosomes	Phospholipid, ethanol (high concentration up to 20–50% *w/w*)		Ethanol increases the fluidity of phospholipid bilayers and disrupts the membrane barrier of SC.	[17]
	Transfersomes	Phospholipid, edge activators		The edge activators increase the flexibility and deformability for passing through the narrow pores.	[18]
	Niosomes	Nonionic surfactants, cholesterol		Similar to liposome The nonionic surfactants enhance the drug encapsulation efficiency.	[19]
	Glycerosomes	Phospholipid, glycerol, cholesterol		Similar to transfersome The glycerol improves the elasticity and deformability.	[20]
	Invasomes	Phospholipid, ethanol (low concentration as 3% *w/w*), terpenes		Ethanol and terpenes disrupt the SC lipid structure and increase the membrane elasticity.	[21]
Lipid nanoparticles	Solid lipid nanoparticles	Solid lipids	Solid particles with non-aqueous core	The lipid nanoparticles form a mono-layer lipid film to enlarge the inter-keratinocyte gap. The lipid components and incorporated surfactant could disrupt skin structure and increase the intercellular space.	[11,22]
	Nanostructured lipid carriers	Solid and liquid lipids			
Polymeric nanoparticles		Natural polymers and synthetic polymers	Solid colloidal carriers	They create a drug concentration gradient to enhance the drug permeation. Some small or positively charged polymeric nanoparticles can penetrate through epidermal barrier through paracellular route and hair-follicle route.	[13,22,23]
Inorganic nanoparticles	Gold nanoparticles	Au	Solid and rigid particles	Some small nanoparticles can penetrate the skin through the lipidic matrix of the stratum corneum and through hair follicle orifices.	[24,25]
	Fe_3_O_4_ nanoparticles	Fe_3_O_4_		They penetrate into deeper dermis via transfollicular route.	[26]
	CuS nanoparticles	CuS		The near-infrared absorption induces the localized thermal ablation of SC and facilitate the penetration.	[27]
Dendrimers		Poly (amidoamine) and other polymers	High-branched polymeric nanocarriers	Serving as penetration enhancer through interaction with skin lipid bilayers	[28]
Micelles		Amphiphilic polymers, surfactants	Spherical or irregular monolayer structure	Improving the water solubility of drugs	[29,30,31]
Nanoemulsions		Water, oil, surfactants	Dispersions of water and oil	Disrupting the skin lipid bilayers; Increasing solubility for both liposoluble and water-soluble drugs	[22]
Nanogels		Polymers	Cross-linked network structure	The nanocarrier dispersions prolong the topical contact duration and increase localized drug concentration. Cationically charged nanogels interact with epidermis.	[32,33,34]

**Table 2:** Summary of introduced surface modifications via their functions.

Functions		Surface Modifier		Nanocarrier	Achieved Improvement	Ref.
Enhanced penetration efficiency		Oleic acid		Niosomes; polymeric nanoparticles	Increasing penetration depth	[46,47]
		Ceramides		Vesicular phospholipid system	Increasing drug deposition in the skin	[48]
		Polymers	PEG	PDA NPs	Preventing aggregation; Increasing penetration depth	[49]
			PIPP	Cellulose nanocrystal (CNC)-stabilized emulsions	Increasing surface hydrophobicity and stability; Increasing penetration depth	[50]
		Cell-penetrating peptide		Lipid-based vesicles	Increasing cell uptake and internalization; Improving skin penetration and permeation	[51,52,53]
Controlled release	Sustained drug release	Oleic acid		Polymeric nanoparticles	Controlled release lasting 72 h	[54]
		Dabco surfactants		Liposomal system	Sustained release of 50% loaded drug in 12 h	[55]
		Oxidized chitosan		Nanofibers	Sustained release of 50% loaded drug in 9 h	[56]
	Activated modulated release	Thermosensitive poly-N-isopropylacrylamide (PNIPAAm)		Liposomes	Drug release and skin permeation begin from 37 °C	[57]
		Thermosensitive polyglycerol (tPG)		Nanogels	Drug release and skin permeation begin from 40 °C	[58]
		Cationic hydroxyethyl cellulose (JR400)		MoS_2_ nanoparticles	Irritated by near-infrared 808 nm laser	[59]
Targeting drug delivery		Hyaluronic acid and derivatives		Niosomes; Cdot-Ce6	Cancer cell targeting	[60,61]
		Epidermal growth factor		Fusion peptide carrier	Melanoma cell targeting	[62]
		BQ-788 (endothelin ETB receptor ligand)		ZnO quantum dots	Melanocyte targeting	[63]
		Integrin αvβ3 ligand		*Escherichia coli* derived outer membrane vesicles	Melanoma cell targeting	[64]
